# Radiation nanosensitizers in cancer therapy—From preclinical discoveries to the outcomes of early clinical trials

**DOI:** 10.1002/btm2.10256

**Published:** 2021-09-23

**Authors:** Colette Bilynsky, Nadine Millot, Anne‐Laure Papa

**Affiliations:** ^1^ Department of Biomedical Engineering The George Washington University Washington District of Columbia USA; ^2^ Laboratoire Interdisciplinaire Carnot de Bourgogne UMR 6303, CNRS, Université Bourgogne Franche‐Comté Dijon Cedex France; ^3^ Present address: Department of Biomedical Engineering Carnegie Mellon University Pittsburgh Pennsylvania USA

**Keywords:** cancer, nanosized radiosensitizers, radiotherapy

## Abstract

Improving the efficacy and spatial targeting of radiation therapy while sparing surrounding normal tissues has been a guiding principle for its use in cancer therapy. Nanotechnologies have shown considerable growth in terms of innovation and the development of new therapeutic approaches, particularly as radiosensitizers. The aim of this study was to systematically review how nanoparticles (NPs) are used to enhance the radiotherapeutic effect, including preclinical and clinical studies. Clinicaltrials.gov was used to perform the search using the following terms: radiation, cancer, and NPs. In this review, we describe the various designs of nano‐radioenhancers, the rationale for using such technology, as well as their chemical and biological effects. Human trials are then discussed with an emphasis on their design and detailed clinical outcomes.

## 1 HISTORY OF RADIATION IN CANCER

The first medical X‐ray image of a hand is attributed to German physicist Wilhelm Conrad Röntgen in 1895. In the years to follow, it became clear that the use of X‐rays was associated with side effects such as skin burns^1^ and hair loss.^2^ Thus, the idea of using them as a potential mean of treating tumors emerged. In parallel, there have been many advances in the development of X‐ray tubes.^3^ X‐rays are produced from electrical current, that is, an electron beam discharged from a hot cathode in vacuum. The beam of electrons produces X‐rays as it encounters the anode or glass wall (in the case of the early‐developed X‐ray tubes, e.g., the Coolidge tube, in 1913). The first reported case of using radiation therapy to treat cancer with a similar device was reported in 1896 by French Physician Dr. Victor Despeignes for the exploratory treatment of a patient with gastric cancer.^4^, ^5^ Then, the first instance of brachytherapy shortly followed Drs. Pierre and Marie Curie's discovery of radium in 1898. In July 1903, Dr. Alexander Graham Bell suggested the use of brachytherapy in a letter to Dr. Z. T. Sowers; the correspondence was published in the journal *Nature*.^6^ In October 1903, Dr. Margaret Abigail Cleaves, an American physician focused on psychological and gynecological disorders, was the first to use radium in the treatment of gynecologic malignancies via brachytherapy.^7^ Since then, the management of cancer patients using radiation has significantly evolved, with the greater understanding of the physics and adverse events (AEs) that accompany X‐ray therapy. Indeed, radiation‐induced leukemia or malignant skin changes were evidenced as early as 1900.^8^ Optimization of the dose intensity delivered and its fractionation have been some of the major improvements, along with the deployment of modalities that have significantly improved the spatial targeting of radiation.^9^, ^10^ Over the last few years, conventional radiotherapy has progressively been replaced by conformal radiotherapy (CFRT) where the radiation beam is geometrically controlled to fit the tumor shape while sparing the surrounding organs. Additionally, intensity‐modulated radiation therapy (IMRT) has become the gold standard in radiotherapy treatment by allowing a controlled irradiation field shape, like conventionally fractionated radiation therapy (CFRT), but its intensity is also modulated within the irradiated area. Today, IMRT is associated with image‐guided radiotherapy and radiotherapy, in general, is now a common component of multidisciplinary cancer care, used in conjunction with surgery and other systemic therapies.^9^

More recently, a new irradiation modality called hadron therapy has been developed, using charged particles instead of photons.^10^ In 1946, Robert R. Wilson was the first to propose the use of proton beams for the treatment of cancer.^11^ The major advantage of this technique is its depth‐dose profile characterized by a significant increase in the dose deposited at the end of the particle path.^12^ Based on clinical data and in vivo experiments, Paganetti et al. have demonstrated that the relative biological effectiveness (RBE) of protons was close to 1.1. This implies that protons lead to the same cancer cell death in comparison to photons, with a 10% reduced dose of radiation delivered to tissues.^13^ RBE depends on several parameters including the linear energy transfer (LET), which is why the RBE of charged particles varies along their path and is significantly higher near the Bragg peak. Photons have a low LET coefficient, meaning that they ionize atoms in the tissue that are spaced by several tenths of a micrometer apart, sparsely and randomly along their path. In contrast, LET is higher for protons, which generate more radicals per particle track than lower‐LET ones.^14^ Finally, hadron therapy relies on two key parameters^15^: (i) ballistic features allowing a dose optimization into tumor volume, sparing surrounding healthy tissues, and (ii) biological features that allow a greater RBE associated with a high LET along their path. Using this method, the dose can be deposited in a chosen area with a high accuracy. Proton therapy is an especially good option for the radiation therapy of pediatric cancers, given the sensitivity of developing organs to radiation and potential long‐term side effects.^16^

## 2 INTERACTION OF PHOTONS WITH MATTER

The photon attenuation in matter is due to three different processes: photoelectric effect, Compton scattering (Rayleigh scattering can be neglected), and pair production.^17^ A photon can transfer its energy to one electron of the target leading to an electron ejection from the atom, that is, the photoelectric effect. During this effect, the energy of the incident photon is totally transferred to an electron in an inner shell (photoelectron). The vacancy created in the inner layer is filled by an electron from an outer layer, the energy being released in the form of a fluorescence X‐ray photon or an Auger electron. For K shell vacancies, the Auger yield decreases with atomic number (Z), and for Z = 30 (zinc) the probabilities of the emission of X rays from the innermost shell and of the emission of Auger electrons is about equal.^18^ When Compton scattering occurs, the electron is spread away together with a new photon that has a lower energy than the incoming one. Highly energetic photons (E > 1.02 MeV) produce an electron‐positron pair. This positron slows down in matter and strikes with an electron leading to the formation of two gamma rays of 0.511 MeV. Photons are produced in opposite directions. Depending on the atomic number of the material, each phenomenon contributes in a different way to the total photon attenuation. At low energy (below 100 keV), the photoelectric effect governs the attenuation. For energies between 100 keV and 10 MeV, the Compton effect becomes the primary process for attenuation, regardless of the intervening material.^19^ Above 10 MeV, pair production dominates. Since energies used in clinical practice are usually between 0.3 and 20 MeV, the Compton effect is the dominant effect when the photon passes through tissues.

## 3 PRECLINICAL STUDIES BASED ON THE USE OF NANO‐SENSITIZERS IN CANCER RADIATION THERAPY

### 3.1 Rationale for using NPs to target tumors and initial evidence of radiation enhancing property of NPs

Tumor growth is associated with angiogenesis, the formation of a vascular bed surrounding the tumor, in order to provide it with essential nutrients that support continued cancer growth. The newly formed vasculature is in essence disorganized and leaky, which provides an ideal environment for nanosized particles injected in the bloodstream to passively permeate into tumor tissue. This effect has been named the enhanced permeability and retention (EPR) effect.^20^ Nanoparticles (NPs) end up at the tumor site due to the porosity of the local endothelium and are subsequently retained in the tumor because of the ineffective lymphatic drainage at the tumor level.^20^ Thus, NPs represent an ideal candidate to deliver chemotherapeutic drugs to tumor site, minimizing side effects to healthy tissues. Even though some nanomedicines have reduced toxicity for patients compared to free drug (e.g., Doxil vs. Doxorubicin), their accumulation in the tumor usually represents a very limited fraction of the injected dose.^21^ Indeed, Wilhelm et al. reviewed 10 years of data and concluded that 0.7% of the injected dose typically reaches the tumor site.^21^ Thus, strategies have been developed for particles to actively target the tumor site by tagging them with molecules able to recognize ligands within the peripheral vascular bed (e.g., vascular endothelial growth factor (VEGF) receptor^22^), the extracellular matrix^23^ or on the surface of cancer cells (e.g., folate receptor,^24^ epidermal growth factor receptor (EGFR), human epidermal growth factor receptor 2 (HER2)^25^, ^26^). Interestingly, tumor homing can also be enhanced by utilizing tubular‐shaped NPs,^25^ which undergo high levels of phagocytosis by immune cells compared to their spherical counterparts. These cells subsequently travel to the tumor via the bloodstream, delivering the nanotubes and their drug payloads. Additionally, tagging NP surface with the marker of self (CD47) delays their recognition and clearance, thus increasing their ability to accumulate at tumor site.^26^ Similar strategies based on coating the NPs with RBC (red blood cell)^27^ or leukocyte^28^ membranes have successfully extended the circulation time of NPs in murine models. Using external stimuli to improve targeting has also been explored, such as using ultrasound stimulation to break nanoassembled microparticles, exclusively at tumor site, thus enhancing their accumulation.^29^ Targeted delivery using an external magnetic field is also possible; iron oxide NPs represent an efficient means of drug delivery in that case.^30^ Hyperthermia or ultrasound have also been used to break biological barriers at microscopic level (e.g., blood–brain barrier [BBB]) and improve nanomedicine delivery^30^ (Table 1).

**TABLE 1:** Summary of the cited pre‐clinical studies involving NP radiosensitizers

Authors (year)	Nanoparticle	Animal model	Route of administration	Radiation dose	Results
Hainfeld et al. (2004)^58^	1.9 nm gold NPs	Mice with subcutaneous EMT‐6 mammary carcinoma	Intravenous injection	250 kVp X‐rays	Mice treated with NPs and radiation had a 86% one‐year survival (20% radiation alone, 0% NP alone)
Zhang et al. (2014)^61^	54 nm wide Bi_2_Se_3_ nanoplates protected with poly(vunylprollidone)	Nude mice with U14 tumors (cervical carcinoma)	Intraperitoneal injection	^137^Cs γ‐radiation of 3600 Ci, 5 Gy, 662 keV	After 25 days, tumors in mice treated with NPs and radiation grew 1.47 times (3.2 times with NPs alone, 2.4 times with radiation alone)
Yong et al. (2016)^65^	3.5 nm GdW_10_ nanoclusters (NCs) functionalized with BSA	BALB/c female mice with subcutaneous BEL‐7402 tumor (hepatocellular carcinoma)	Intratumoral injection	6 Gy, X‐rays	After 18 days, mice treated with NCs and radiation showed a relative tumor volume change (V/V_0_) of ~1 (~8 with NCs alone, ~4 with radiation alone)
Lux et al. (2019)^66^	AGuIX (polysiloxane Gd‐chelates based NPs)	Rats with 9LGS (gliosarcoma)	Intravenous injection	6 MV (clinical irradiator)	Mean survival time in rats treated with AGuIX NPs and irradiation was 72.9 ± 35.5 days (26 ± 0.5 days for controls, 39 ± 0.5 days for irradiation alone)
		Mice with melanoma B16F10 brain metastases	Intravenous injection	7 Gy (320 kV preclinical irradiator)	Mean survival time in mice treated with AGuIX NPs and irradiation was 15 days (12 for controls, 13 days for irradiation alone)
		Mice with capan‐1 tumors (pancreatic cancer)	Intravenous injection	10 Gy (220 kV preclinical irradiator)	Mean survival time in mice treated with AGuIX NPs and irradiation was 60 days (13 for controls, 35 days for irradiation alone)
		Mice with capan‐1 tumors (pancreatic cancer)	Intravenous injection	10 Gy (6 MV clinical irradiator)	Mean survival time in mice treated with AGuIX NPs and irradiation was 93 days (30 for controls, 60 days for irradiation alone)
		Mice with subcutaneous A375 tumor (melanoma)	Intratumoral injection	10 Gy (220 kV preclinical irradiator)	25 days after treatment tumor volume increased by 3% in mice treated with NPs and irradiation (82% for irradiation alone)
		Mice with subcutaneous SQ20B tumor (head and neck cancer)	Intratumoral injection	10 Gy (320 kV preclinical irradiator)	By end of week 7, mean tumor growth for the mice treated with NPs and irradiation was 5 times smaller than the irradiated only group and 11 times smaller than control group
		Rats with xenografted radioresistant chondrosarcoma (SWARM)	Intratumoral injection	4 Gy irradiation	Rats treated with NPs along with irradiation had a longer average survival time versus irradiation alone
		Mice with H358‐Luc orthotopic lung tumor (luciferase‐modified non‐small cell lung cancer)	Nebulized through airways	10 Gy (220 kV preclinical irradiator)	Mean survival time in mice treated with AGuIX NPs and irradiation was 112 days (83 for controls, 77 days for irradiation alone)
		Nude mice with HepG2 tumor (hepatocellular carcinoma)	Intraperitoneal injection	6 Gy (250 kV irradiator)	Found that 10 mg of NPs along with irradiation can suppress glucose metabolism in the tumor
	Bi@AGuIX (polysiloxane Gd‐chelates based NPs with bismuth)	Mice with A549 tumors (lung adenocarcinoma)	Intravenous injection	10 Gy (6 MV irradiator)	100% of mice treated with irradiation and NPs survived 90 days (control groups survived 70 days, irradiated groups survived 80 days)
Maggiorella et al. (2012)^67^	NBTXR3 (hafnium oxide NPs)	Nude NMRI mice with xenografted HT1080 tumors (fibrosarcoma)	Intratumoral injection	0, 4, 8 Gy (^60^Co source)	The NPs showed a mean dose enhancement of the radiation above 1.5 at 4 and 8 Gy
		Nude NMRI mice with xenografted A673 tumors (Ewing's sarcoma)	Intratumoral injection	15 Gy (^60^Co source)	Mice treated with NPs and radiation had tumor growth inhibition of 82% (72% for radiation alone)
		Nude SWISS mice with xenografted HCT116 tumors (colorectal carcinoma)	Intratumoral injection	2 × 4 Gy or 1 × 8 Gy (iridium‐192 source)	Complete tumor response in both cases (2 × 4 Gy or 8 Gy). Mice treated with radiation and NPs had significantly higher survival percentages than those treated with radiation or NPs alone in both cases.
Mirjolet et al. (2017)^70^	Titanate nanotubes loaded with docetaxel (DTX)	Nude Balb/c mice with PC‐3 tumor (prostate adenocarcinoma)	Intratumoral injection	3 × 4 Gy (brachytherapy iridium projector)	The tumors of mice treated with radiation and NTs reached 1000 mm^3^ in 73.7 days (56 days with free DTX and radiation, 40.5 days NTs, 30.8 days free DTX)
Loiseau et al. (2019)^73^	Titanate nanotubes loaded with docetaxel and gold NPs (TiONts‐AuNPs‐PEG_3000_‐DTX)	Nude Balb/c mice with PC‐3 tumor (prostate adenocarcinoma)	Intratumoral injection	3 × 4 Gy	The tumors of mice treated with radiation and TiONts‐AuNPs‐PEG_3000_‐DTX reached 1000 mm^3^ in 55.2 days (49.9 days for TiONts‐DTX with radiation, 39 days for TiONts‐AuNPs‐PEG_3000_‐DTX, 40.67 days for TiONts‐DTX, 35.8 days for control)
Zhang et al. (2011)^75^	Liposomal cisplatin	C57BL/6N mice with a Lewis lung carcinoma tumor implanted in right flank	Intravenous injection	2, 6, 16, or 28 Gy	Mice treated with NPs and radiation had a significantly longer tumor growth delay compared with those treated with radiation alone, or cisplatin along with radiation
Davidi et al. (2017)^76^	20 nm gold NPs coated with glucose and cisplatin	Nude mice with subcutaneous A431 tumor (head and neck cancer)	Intravenous injection	6 MV	The final tumor volume (24 days) of mice treated with NPs and radiation was 50% of the original volume (150% for radiation alone, 110% for free cisplatin and radiation)
Werner et al. (2011)^82^	70 nm PLGA particle encapsulating docetaxel functionalized with folate	Mice with xenografted KB tumor	Intravenous injection	12 Gy	Mice treated with the folate NPs and radiation showed a significant tumor growth delay compared to untargeted NPs
Cui et al. (2014)^84^	Gelatinase‐cleavage peptide with poly(ehylene glycol) and poly(□‐caprolactone) polymer NPs encapsulating docetaxel	Nude BALB/c mice with subcutaneous xenografted BGC823 tumors (gastric cancer cells)	Intravenous injection	3 × 5 Gy (4 MeV electron beam)	After 28 days, mice treated with both the NPs and radiation had the smallest tumors compared to those treated with free docetaxel with or without radiation, radiation alone, and NPs alone
Zhang et al. (2019)^86^	Doxorubicin‐ and tetrahydrocurcumin‐loaded and transferrin‐modified PEG‐PLGA NPs	Nude mice with xenografted subcutaneous C6 tumors (glioma)	Intravenous injection	5 × 3 Gy	Mice treated with NPs and radiation had the lowest tumor volume of any other group (radiation alone, radiation and free tetrahydrocurcumin, radiation with free doxorubicin, radiation, and both free drugs)
Zong et al. (2019)^86^	Angiopep‐2‐modified lipid‐poly (hypoxic radiosensitized polyprodrug) NPs loaded with temozolomide	Mice with intracranial C6 tumors (glioma)	Intravenous injection	3 × 2 Gy (0.3 Gy/min)	Mice treated with NPs and radiation had the longest median survival time of 67 days (28.5 days for the control, 32 days for the radiation alone)
Xu et al. (2016)^91^	Liposomal perfluorohexane	Balb/c mice with CT26 tumor (colon cancer)	Intravenous injection	5, 7.5, 10 Gy (1.25 Gy/min, 6 MV X‐rays)	Mice treated with NPs and radiation (7.5 Gy and 10 Gy) had significantly increased tumor growth time (time for the tumor to grow 10‐fold) compared with the control
Johnson et al. (2017)^92^	Dodecafluoropentane nanoemulsion	SCID mice with xenografted subcutaneous Hs‐766 T (pancreatic cancer)	Intravenous injection	12 Gy (^60^Co source)	Mice treated with NPs and radiation showed a tumor growth increase of 2% per day (the control group had a rate of 50% per day)
Jeong et al. (2009)^95^	Gold NPs loaded with β‐lapachone with anti‐EGFR targeting ligand	BALB/c‐nu mice with xenografted A549 tumor (lung carcinoma)	Intravenous injection	5 Gy (6 MV photon beam)	Mice treated with NPs and radiation showed a final tumor size of 29% (control was 100%, NPs alone was 71%, radiation alone was 57%)
Bouras et al. (2015)^96^	Iron‐oxide NPs with anti‐EGFR targeting ligand	Mice with xenografted EGFRvIII‐expressing U87MG (glioblastoma)	Convection‐enhanced delivery	2 × 10 Gy	Mice treated with NPs and radiation showed a significant survival benefit compared to the group treated with the anti‐EGFR antibody and radiation, and the control group
Chattopadhyay et al. (2013)^101^	Gold NPs with anti‐HER2 targeting ligand	Athymic CD1 nu/nu mice with a sustained release 17β‐estradiol pellet with xenografted MDA‐MB‐361 tumor (breast cancer)	Intratumoral injection	11 Gy, 100 kVp X‐rays	Mice treated with NPs and radiation showed end tumor size (at 4 months) to be 46% of the original (radiation alone showed an increase in size of 11%)
Liu et al. (2017)^103^	Gold and iodine NPs with anti‐RhoJ targeting ligand	BALB/c nu/nu nude mice with patient‐derived xenografted tumor (ER^+^, HER‐2^−^, PR^−^)	Intravenous injection	5 Gy	Mice treated with NPs and radiation showed no death over the observation period of 56 days. The first mice died on days 14 and 16 in the control and radiation alone groups
Kefayat et al. (2019)^106^	Albumin‐stabilized gold NPs with targeting agent (folic acid, glucose, or glutamine)	BALB/c mice with 4 T1 tumor (Breast Cancer)	Intravenous injection	6 Gy (6 MV X‐rays)	Radiation with NPs decorated with glutamine or folic acid showed the best tumor growth inhibition and were significantly more effective than radiation alone or undecorated NPs and radiation
Kefayat et al. (2019)^111^	Folic acid and BSA decorated gold nanoclusters	Wistar rats with an intracranial C6 tumor (glioma)	Intravenous injection	6 Gy	Mice treated with NPs and radiation had a mean survival time of 25.0 ± 1.5 days (control had 12.8 ± 0.7 days, NPs alone had 13.1 ± 0.7 days, radiation alone had 18.3 ± 1.0 days)
Zhang et al. (2018)^112^	Polythylenimine linked by *β*‐cyclodextrin NPs both functionalized with folic acid and loaded with plasmid HGFK1	BALB/c‐nu/nu mice with subcutaneous xenografted U87 tumor (Glioma)	Peritumoral injection	3 × 3 Gy	Mice treated with NPs and radiation had a median survival time of 78 days (control was 53 days, NPs alone was 64 days, radiation alone was 65 days)
Su et al. (2015)^114^	Gold NPs with cRGD and radioactive iodine‐125	BALB/c mice with subcutaneous NCI‐H466 tumor (small cell lung cancer)	Intravenous injection	5 Gy (^60^Co source)	Mice treated with NPs and radiation had a volume increase of 15.2% ± 17.8% (radiation alone was 137.1% ± 35.5%, control was 312.1% ± 96.9%)
Liang et al. (2017)^115^	Gold nanoclusters with cRGD peptide	Mice with 4 T1 tumor (breast cancer)	Intravenous injection	6 Gy (160 kVp irradiator)	The weight of the tumors in mice treated with NPs and radiation after 14 days was significantly lower than the control group, radiation alone group, or NPs alone group
Zhao et al. (2016)^116^	RGD‐conjugated silica‐encapsulated gold nanorods	Nude mice with orthoptopic MDA‐MB‐231 tumor (triple negative breast cancer)	Intravenous injection	10 Gy	Mice treated with radiation and NRs had reduced tumor growth (569 ± 154 mm^3^) compared with untargeted NPs (1073 ± 205 mm^3^), radiation alone (1302 ± 261 mm^3^), and the control (1615 ± 303 mm^3^)
Yang et al. (2016)^118^	29 nm RGD‐conjugated gold NPs	Mice with H1299 tumors (NSCLC)	Intravenous injection	10 Gy of γ‐ray radiation	Mice treated with radiation and NPs showed a tumor volume increase of ~43% (control had ~472%, radiation alone had ~310%)
Hua et al. (2018)^122^	Hypoxia‐responsive lipid‐poly‐(hypoxic radiosensitized polyprodrug) NPs	ICR mice with intracranial xenografted C6 tumor (glioma)	Intravenous injection	2 Gy (0.3 Gy/min, 6 MV X‐rays)	Mice treated with NPs and radiation significantly inhibited glioma growth compared with untargeted NPs with radiation, as well as the PBS control
Lee et al. (2020)^81^	pH‐sensitive ECO (1‐aminoethylimino[bis(*N*‐oleoylcysteinylaminoethyl) propionamide]) NPs functionalized with RGD‐PEG loaded with siRNA targeting ATM (ataxia telangiectasia mutated)	Mice with orthotopic U251 tumors (glioblastoma)	Intratumoral injection	3 × 2 Gy	Mice treated with NPs and radiation had an increase in median survival of 19 days beyond the ethanol control group (delivery of siRNA increased survival by 2 days, NPs alone by 7 days, radiation alone by 9 days)
Kievit et al. (2017)^80^	NPs with a superparamagnetic iron oxide core coated with copolymer of chitosan, PEG, and polyethyleneimine delivering anti‐Ape1 siRNA functionalized with chlorotoxin	Genetic mouse model of glioblastoma	Intravenous injection	5 × 2 Gy (^137^Cs‐γ‐rays, 1 Gy/min)	Mice treated with NPs and radiotherapy increased overall survival time by 50 days, double the increase found with the addition of radiotherapy alone
Van de Ven et al. (2017)^78^	Lipid‐based nanoformulation of olaparib	Nude (nu/nu) mice with subcutaneous FKO1 tumor (radiation‐resistant prostate cancer)	Intravenous injection	10 Gy (X‐rays)	Mice treated with NPs and radiation showed the slowest tumor growth of 48 days to 1500 mm^3^ which was greater than the untreated group or those treated with radiation of NPs alone

Besides utilizing active targeting, local administration of NPs can allow for increased accumulation of the radiosensitizers at the tumor site, while limiting the potential toxicity associated with systemic circulation.^31^ Hence, the recent review by Boateng and Ngwa^31^ highlights the various possible routes of administration of nano‐radioenhancers from passive delivery systems to implantable‐sustained release systems. Utilizing “smart” spacers (i.e., implantable release systems) during radiotherapy allows for NP radioenhancers to be locally applied.^32^ As spacers are often used during radiotherapy to guide the geometric localization of the treatment, switching from inert spacers to “smart” spacers would not require additional procedures.^32^ Inhalation of radiosensitizers also allows for local administration to the lungs and has been shown to be effective.^33^ Lastly, as the liver and spleen are the primary clearance organs of such biomaterials, strategies consisting of priming/saturating the liver with ghost particles before administering the nanomedicine have been successfully achieved.^34^ The parameters affecting biodistribution, including circulation time following IV injection of NPs, are highly dependent on NP's intrinsic properties such as their size, composition, surface charge, surface functionalization (along with grafting density), shape, etc.^35^ In addition, NP interaction with plasma proteins leads to the formation of an adsorbed protein corona that further modulates NP fate as it can affect its resulting size, surface charge, and overall stability.^35^, ^36^ PEGylation of the NP surface is a widely used strategy to significantly decrease plasma protein adsorption and improve circulation time of NPs.^37^

**FIGURE 1:**
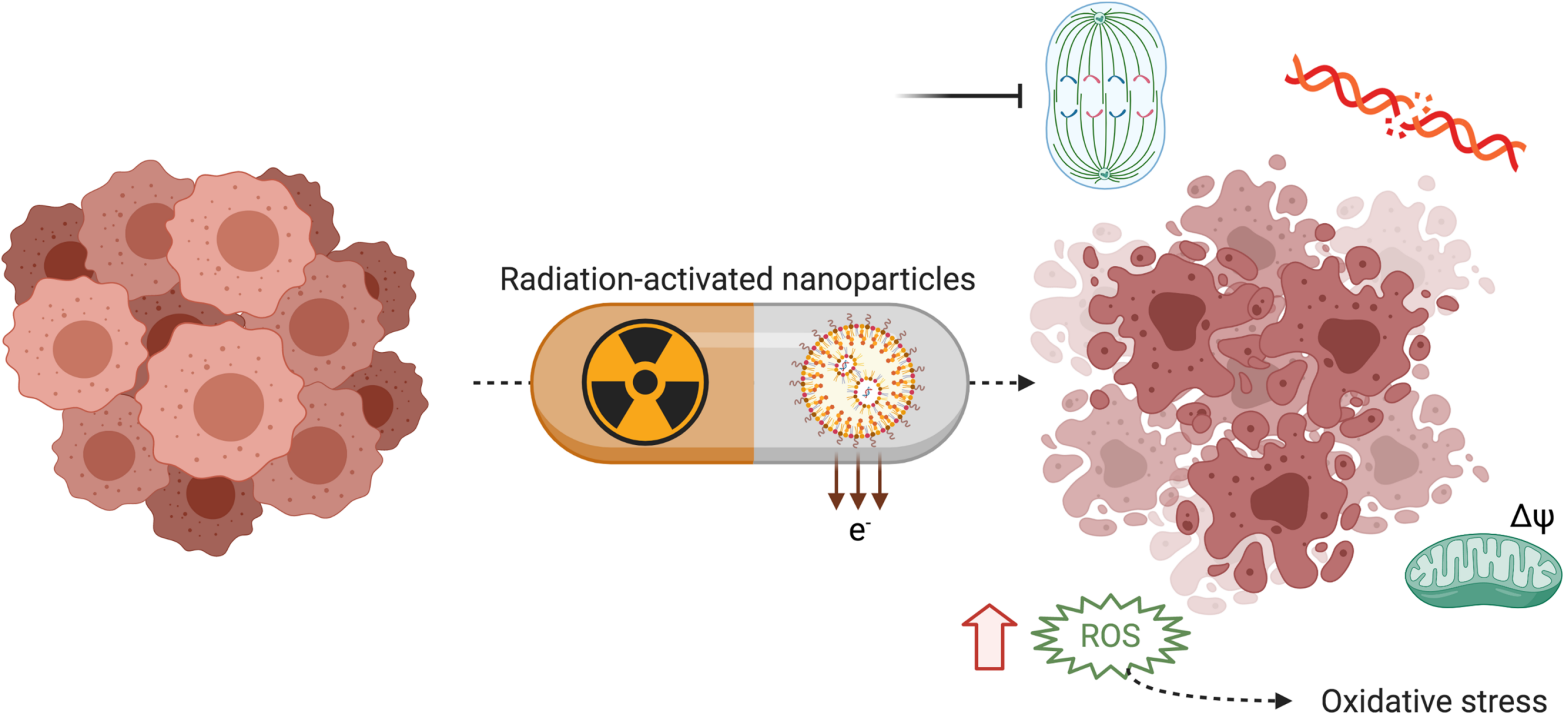
Schematic of the radiobiological effects of nanoparticle radiosensitizers. Radiation‐activated NPs, or nano‐radioenhancers, enable sensitization of tumor cells to radiation by the synergistic production of reactive oxygen species (ROS) inducing oxidative stress, cell cycle arrest, and DNA double strand damage, and ultimately, cell death. Mitochondrial oxidation involvement has been highlighted in some studies as well.^44^, ^45^ Created with BioRender.com

Nano‐radioenhancers are capable of enhancing the sensitivity of cancer cells to radiation. The biological effects subsequent to the use of nano‐enhancers to potentiate tumor cell radiation have been reviewed by Sun et al.^38^ and include: (i) reactive oxygen species (ROS) production (leading to oxidative stress, also called the chemical phase^39^), as well as (ii) DNA damage. Both the levels of ROS production and DNA damage appear to be inversely proportional to the size of NPs,^38^ suggesting that particles with larger surface area to volume ratios produce more ROS and DNA damage. Next, nano‐sensitizer‐assisted radiation also induces (iii) cell cycle arrest in the G2/M transition,^40^, ^41^, ^42^ which then leads to apoptosis (Figure 1). However, cell cycle arrest in G1/S followed by senescence has also been observed with nanodiamonds.^43^ Mitochondrial involvement has been highlighted in multiple studies.^44^, ^45^ For example, a study by Ghita et al. used soft X‐ray microbeam (carbon K‐shell, 278 eV) to achieve subcellular targeting of radiation (i.e., cytoplasmic and nuclear irradiation) in an effort to better understand the mechanistic effects of gold nano‐radioenhancers.^44^ Exclusive cytoplasmic irradiation of MDA‐MB‐231 human breast cancer cells combined with 1.9 nm Aurovist™ (located in the cytoplasm) still led to DNA damage, along with mitochondrial depolarization (oxidation). Ghita et al. highlighted that modulation of the physico‐chemical parameters of the particles might lead to different effects.^44^ Many studies describe the NP's ability to enhance cell death upon irradiation. Interestingly, some papers report significant radiosensitization effects in vitro with very low quantity of NPs.^46^ Regarding this study,^46^ Penninckx et al. calculated that 0.001% of gold NPs per mass (650 NPs per HepG2 human liver cancer cells) led to a radiosentizing effect which was 250 times greater than predicted.^39^ This highlights that critical factors for radioenhancement go beyond the dose of NPs.^47^ Oxidative stress has been highlighted as playing a key role as demonstrated by Daems et al. and Penninckx et al. They found that gold NPs inhibit thioredoxin reductase (TrxR) and glutathione reductase, regulators of redox reactions, in both cancer and normal cells.^47^, ^48^ Guerreiro et al. introduce the idea of the catalytic nature of NPs, with “a sea” of radiolytic reactants near the NP surface influencing the radioenhancer capability.^49^ This point of view may explain why some NPs made of elements with relatively low atomic numbers are also efficient as radiosensitizers. Lastly, a literature examination by Dr. Kempson focusing on the mechanisms involved in nanoradiosensitization highlights that nanosensitizer effects cannot be generalized as they depend on both the biological environment and particle intrinsic properties.^50^ Another important parameter has been highlighted by Cui et al., who demonstrated that the radiosensitization of gastric cancer cells with miRNA‐200c delivery via PEG‐Pep‐PCL copolymers NPs may be explained by a decrease in invasiveness and better targeting to radioresistant cancer stem cells.^51^

**FIGURE 2:**
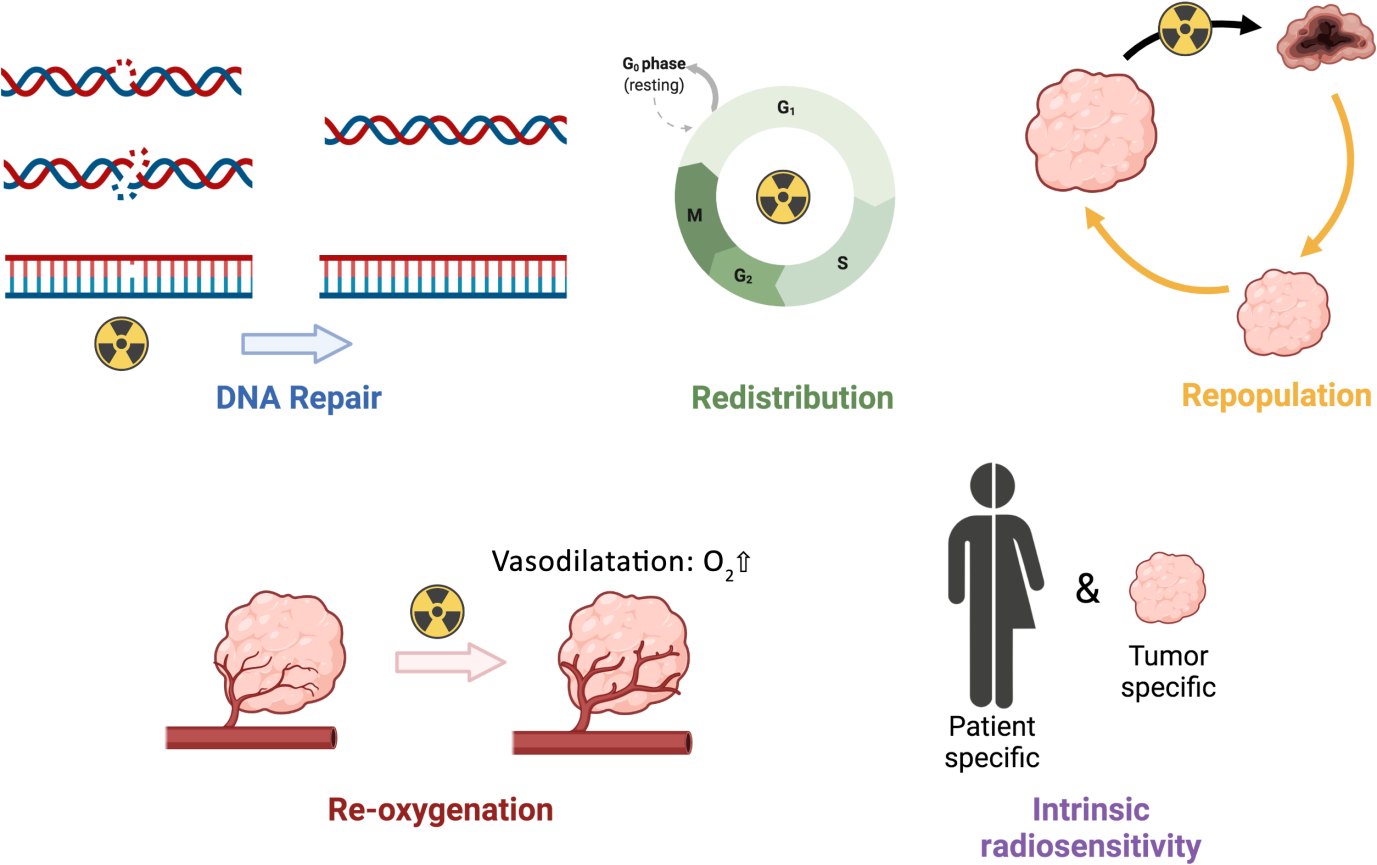
Schematic of the 5R's of radiotherapy: DNA repair, redistribution, repopulation, re‐oxygenation, and intrinsic radiosensitivity. Created with BioRender.com

Finally, five factors have been identified to explain the biological effects of radiotherapy, referred to as the “5 R's of radiotherapy”^39^ (Figure 2): Repair (DNA repair processes after irradiation), Redistribution (among the various cell cycle phases), Repopulation (cellular growth and proliferation between radiotherapy fractions), Re‐oxygenation (radiation induces vasodilatation that enables increased tissue perfusion^52^; the increased oxygen concentrations in the tumor microenvironment facilitate two effects during the subsequent radiotherapy fraction: ROS production and cell death, while cells in the hypoxic fraction of the tumor remain more resistant to the treatment) and intrinsic radiosensitivity (tumor and/or patient‐specific response). As previously mentioned with several examples, NPs may synergize with several of these factors. In a recent review, Penninckx et al. summarize how gold NPs, used as radioenhancers, affect these 5‐R factors at the molecular and cellular levels.^39^ In this review, the authors also point out the major differences induced in these factors for low LET radiation (mainly X‐rays) and high LET radiation (i.e., protons, alpha rays, or heavy ions) when combined with nano‐radioenhancers. In particular, they notice that, independent of the proton energy or the gold NP size/concentration, the physical enhancement is negligible, even if significant radiosensitization effects are observed.^53^ Moreover, Heuskin et al. calculated that the interaction probability of gold NPs with proton radiations is negligible; demonstrating that chemical or biological enhancement should be envisioned.^54^ Interestingly, the oxygen‐enhancement ratio (OER, defined as the ratio of hypoxic over normoxic doses of radiation leading to the same effect) decreases with increasing LET.^55^ For example, Barendsen et al. demonstrated that higher LET α‐particles have an ability to affect hypoxic and normoxic T1g cells to a similar level given an OER close to 1 (i.e., 1.3) at optimum LET (around 100 keV/μm).^55^

### 3.2 NPs with intrinsic radioenhancer properties

The ability of NPs to act as X‐ray radiosensitizers is commonly explained via the physical phenomena described in Section 2, and particularly due to the increased absorption of X‐rays associated with the emission of secondary electrons and fluorescence photons.^56^ These phenomena lead to an enhancement of energy deposition. Thus, in the context of radio‐enhancement, priority has been given to high atomic number elements. The combination of photon radiation and heavy NPs leads to local radiation hardening and higher LET.^57^ Among them, gold (Z = 79) has been widely studied for radiation therapy due to its biocompatibility.^58^, ^59^ Hainfeld et al.^58^ demonstrated that gold NPs injected in mice bearing subcutaneous EMT‐6 mammary carcinomas induce a one‐year survival of 86% versus 20% with X‐rays alone. Bismuth (Z = 83) and platinum (Z = 78) have also been investigated to enhance radiotherapy. Bismuth oxide NPs and bismuth selenide nanoplates demonstrate dose enhancements in vitro and in vivo.^60^, ^61^ Li et al. found that ultra‐small platinum NPs (1.7 nm) amplify gamma ray radiation effects by more than 40%, with the most radioresistant organism ever reported (*Deinococcus radiodurans*).^62^

Magnetic NPs have been also evaluated as potent radiosensitizers for the enhancement of radiotherapy, often in combination with hyperthermia. Iron oxide NPs incubated with prostate carcinoma cells led to a dose enhancement of radiotherapy, along with a drastic increase in the concentration of ROS.^63^, ^64^

Gadolinium (Z = 64)‐based NPs are another promising nanoradiosensitizer. Gadolinium nanoclusters have been developed as multifunctional theranostic agents (computed tomography [CT] or magnetic resonance imaging [MRI] imaging coupled with photothermal or radiation therapy) against tumors. They are highly effective in vitro for photothermal ablation of cancer cells and in vivo for radiotherapy of tumors.^65^ Lux et al. have extensively studied an ultrasmall formulation made of polysiloxane and gadolinium chelates, called AGuIX for “Activation and Guidance of Irradiation by X‐Ray.” The gadolinium doubles as a contrast agent along with a radiosensitizer.^66^ Their efficacy as a radiosensitizer has been demonstrated in vitro on multiple cell lines, including a radioresistant head and neck squamous cell carcinoma line. In vivo models have shown AGuIX's ability to radiosensitize multiple types of cancer: glioblastoma (administration route: IV), brain metastases (IV), melanoma (IT), pancreatic cancer (IV), liver cancer (IV), chondrosarcoma (IT), head and neck cancer (IT), and lung cancer (airways).^66^ These preclinical results using the gadolinium particles have led to multiple clinical trials (Table 2), which will be discussed in more detail in Section 4.1.

**TABLE 2:** Clinical trials evaluating the use of radiation nanoenhancers in cancer

Nanoparticle type	Clinical trial short title	Condition	Trial ID, phase, version#	Estimated enrollment^a^	Status^a^	Dates^a^
AGuIX: Polysiloxane Gd‐chelates‐based NPs	Radiosensitization of multiple brain metastases using aguix gadolinium‐based nanoparticles (NANORAD)	Brain metastases	NCT02820454 Phase I v6	15 patients (actual)	Completed	March 2016 to February 2019
	Radiotherapy of multiple brain metastases using AGuIX® (NANORAD2)	Brain metastases	NCT03818386 Phase II v7	100 patients	Recruiting	March 2019 to March 2022
	AGuIX gadolinium‐based nanoparticles in combination with chemoradiation and brachytherapy (NANOCOL)	Gynecologic cancer	NCT03308604 Phase I v2	18 patients	Unknown [previously: recruiting]	May 2018 to May 2021
	Evaluating AGuIX® nanoparticles in combination with stereotactic radiation for brain metastases (NANOSTEREO)	Brain metastases	NCT04094077 Phase II v8	1 patient (actual)	Terminated	January 2020 to February 2021
	Stereotactic brain‐directed radiation with or without AGuIX gadolinium‐based nanoparticles in brain metastases	Brain cancer Brain metastases Melanoma Lung cancer Breast cancer HER2‐positive breast cancer Colorectal cancer Gastrointestinal cancer	NCT04899908 Phase II v1	112 patients	Not yet recruiting	June 2021 to February 2025
	Nano‐SMART: nanoparticles with MR‐guided SBRT in NSCLC and pancreatic cancer	Non‐small cell lung cancer Advanced pancreatic adenocarcinoma Unresectable pancreatic cancer Ductal adenocarcinoma of the pancreas	NCT04789486 Phase I–II v2	100 patients	Recruiting	May 2021 to September 2024
	Reirradiation by nanoparticles and hypofractionated protontherapy of relapses tumors: non‐randomized phase II study. (NANOPRO)	Recurrent cancer	NCT04784221 Phase II v2	46 patients	Not yet recruiting	September 2021 to September 2026
NBTXR3 (PEP503, Hensify®):Hafnium oxide NPs	NBTXR3 crystalline nanoparticles and stereotactic body radiation therapy in the treatment of liver cancers	Liver cancer	NCT02721056 Phase I–II v6	23 patients	Terminated after determination of recommended phase II dose	January 2016 to May 2020
	NBTXR3 nanoparticles and EBRT or EBRT with brachytherapy in the treatment of prostate adenocarcinoma	Prostate cancer	NCT02805894 Phase I–II v14	5 patients (actual)	Terminated due to change in clinical practice	November 2017 to March 2020
	NBTXR3 activated by radiotherapy for patients with advanced cancers treated with an anti‐PD‐1 therapy	Metastasis from malignant tumor of stomach Squamous cell carcinoma of head and neck Metastasis from malignant tumor of cervix Metastatic squamous cell carcinoma Metastasis from malignant melanoma of skin Merkel cell carcinoma Metastasis from malignant tumor of lung Metastasis from malignant tumor of bladder	NCT03589339 Phase I v6	60 patients	Recruiting	January 2019 to March 2023
	NBTXR3 and radiation therapy in treating patients with locally advanced SCC of the oral cavity or oropharynx	Head and neck cancer	NCT01946867 Phase I v6	63 patients	Recruiting	January 2014 to September 2022
	NBTXR3 crystalline nanoparticles and radiation therapy in treating patients with soft tissue sarcoma of the extremity	Soft tissue sarcoma	NCT01433068 Phase I v7	22 patients (actual)	Completed	October 2011 to October 2020
	NBTXR3 crystalline nanoparticles and radiation therapy in treating and randomized patients in two arms with soft tissue sarcoma of the extremity and trunk wall	Soft tissue sarcoma	NCT02379845 Phase II–III v11	180 patients (actual)	Completed and approved (Europe)	March 2015 to September 2020
	NBTXR3 activated by radiation therapy for the treatment of locally advanced or borderline‐resectable pancreatic cancer	Borderline resectable pancreatic adenocarcinoma Locally advanced pancreatic ductal adenocarcinoma Resectable pancreatic ductal adenocarcinoma Stage III pancreatic cancer AJCC v8	NCT04484909 Phase I v2	24 patients	Recruiting	July 2020 to December 2026
	A study of PEP503 with radiotherapy in combination with concurrent chemotherapy for patients with head and neck cancer	Head and neck squamous cell carcinoma	NCT02901483 Phase I–II v6	42 patients	Recruiting	October 2016 to December 2022
	A study of PEP503 (radioenhancer) With radiotherapy and chemotherapy for patients with rectal cancer	Rectal cancer	NCT02465593 Phase I–II v5	42 patients	Recruiting	June 2015 to June 2023
	NBTXR3 and radiation therapy for the treatment of inoperable recurrent non‐small cell lung cancer	Recurrent lung non‐small cell carcinoma Stage I to IIIC lung cancer AJCC v8 Unresectable lung non‐small cell carcinoma	NCT04505267 Phase I v3	24 patients	Recruiting	September 2020 to March 2024
	NBTXR3, chemotherapy, and radiation therapy for the treatment of esophageal cancer	Cervical esophagus adenocarcinoma; Gastroesophageal junction adenocarcinoma Thoracic esophagus adenocarcinoma	NCT04615013 Phase I v3	24 patients	Recruiting	November 2020 to October 2023
	Re‐irradiation with NBTXR3 in combination with pembrolizumab for the treatment of inoperable locoregional recurrent head and neck squamous cell cancer	Recurrent head and neck squamous cell carcinoma Unresectable head and neck squamous cell carcinoma	NCT04834349 Phase II v1	80 patients	Recruiting	March 2021 to May 2025
Ferumoxytol: Iron oxide NPs	Radiotherapy with iron oxide nanoparticles (SPION) on MR‐linac for primary and metastatic hepatic cancers	Hepatic cancers	NCT04682847 Phase I v1	25 patients	Recruiting	November 2020 to December 2022

^a^ Information was collected from Clinicaltrial.gov on June 28, 2021.

Crystalline hafnium (Z = 72) oxide NPs with a hydrodynamic diameter of 50 nm and a negative surface charge (−50 mV) have been injected in radioresistant and radiosensitive human tumor xenografts (mesenchymal and epithelial cell lines, respectively).^67^ The NPs demonstrated marked advantage in terms of survival, tumor growth delay, and local control in both mesenchymal and epithelial human tumor xenografts, when compared with radiation therapy alone. These NPs, called NBTXR3, were well tolerated in animal models. They are now being evaluated in clinical trials which are discussed further in Section 4.2.

Thulium (Z = 69) oxide NPs have been successfully evaluated as CT imaging contrast agents and radiosensitizers in rats bearing 9 L gliosarcomas^68^ and also showed promising in vitro results with patient‐derived cell lines from metastatic cutaneous squamous cell carcinoma.^69^

However, low Z element‐based NPs have also demonstrated significant potential as radiosensitizers. For example, Grall et al. reported that radiation‐exposed hydrogenated nanodiamonds displayed significantly higher ROS compared to both radiation and particle effect alone, as well as DNA damage, cell cycle arrest, and senescence.^43^ Mirjolet et al. used titanate nanotubes to radiosensitize glioblastoma lines (U87‐MG and SNB‐19) and observed a significant production of DNA double strand breaks and cell cycle arrests in the G2/M checkpoint.^41^ A second generation of these nanotubes was then loaded with taxanes to potentiate the radiosensitization effect, as taxanes also promote G2/M arrest, making the cells further susceptible to radiation.^70^, ^71^ In prostate (PC‐3) tumor‐bearing mice, 70% of Docetaxel‐loaded titanate nanotubes were successfully retained in the tumor 7 days after intratumoral (IT) injection and led to significantly reduced tumor growth as early as day 18.^71^ Such nanotubes have also been decorated with iron oxide NPs^72^ and gold NPs,^73^ which could potentially enable their use for both image‐guided and synergistic radiosensitization of tumors.

The lack of systematic evaluation of metal oxide NPs in parallel, led Guerreiro et al. to assess ROS production effect of 22 NP suspensions following 10 Gy of 6‐MV X‐ray photon irradiation.^49^ Key highlights from their ROS production assessment demonstrated that: V_2_O_5_ produced the most hydroxyls upon radiation (further increased in comparison to its production of hydroxyls at baseline, i.e., without radiation, which was already significantly greater than other NPs), while lanthanides did not produce any (both at baseline and with radiation), TiO_2_ showed a trend toward increased superoxide anion production with radiation, MoO_3_ demonstrated a protective effect as highlighted by the decreased superoxide anion level detected compared to water, and the following NPs induced a protective effect regarding singlet oxygen production: V_2_O_5_, NiO, CuO, MoO_3_, (Z between 23 and 42), Nd_2_O_3_, Eu_2_O_3_, Gd_2_O_3_, Dy_2_O_3_ (Z between 60 and 66).^49^ As previously explained, this suggests that surface chemistry of NPs may be the key parameter instead of atomic number. NPs may act as a catalyzer of surrounding chemical reactions and increase the overall concentration of radicals. It is worth noting that these measurements have been done in water and not in biologically relevant conditions.

### 3.3 NPs used to deliver radiation‐enhancer molecules

NPs can also be used as a vehicle to deliver radiosensitizing drugs or molecules to the tumor site rather than the NPs themselves sensitizing the tumor. One common strategy is to deliver chemotherapy to the tumor which can enhance the effects of radiation treatment. Cisplatin, a commonly used chemotherapy, enhances the effects of radiation through its interaction (i.e., high reactivity and electron‐transfer reactions) with the electrons generated during radiation.^74^ Liposomal cisplatin has been found to be effective both in vitro and in vivo against Lewis lung carcinoma. Furthermore, it was found to be more effective as a radiosensitizer than free cisplatin due to increased accumulation within cancer cells.^75^ Cisplatin has also been conjugated to gold NPs for tumor sensitization of head and neck cancers, as well as glioblastoma.^76^, ^77^ This is doubly beneficial as the gold NPs in themselves are radiosensitizers and can act as a tumor imaging agents using CT.^76^

NP formulations that target DNA repair pathways have also shown encouraging results in conjunction with radiation therapy. For example, twice a week administration of NanoOlaparib, a PEGylated lipid based‐NP loaded with the FDA approved PARP (poly[ADP‐ribose] polymerase) inhibitor Olaparib, has demonstrated greater tumor reduction when given along with radiation (focused beam; single dose of 10 Gy) in a prostate tumor model (using murine *Pten* ^pc−/−^;*Trp53* ^pc−/−^ FKO1 cells in a nude mouse model), compared to either radiation or NanoOlaparib controls.^78^ Strategies leveraging NP‐based delivery of PARP (poly[ADP‐ribose] polymerase) inhibitors in conjunction with radiotherapy have been reviewed by Singh et al.^79^ In addition, approaches using NP‐based delivery of siRNA targeting DNA repair proteins have also been developed.^80^, ^81^ For example, Kievit et al. successfully knocked down Ape1 (apurinic endonuclease 1) using iron oxide NPs functionalized with PEG/chitosan/PEI (polyethyleneimine) to deliver siRNA in a genetic glioblastoma mouse model.^80^ Mice received siRNA NPs IV and whole brain gamma radiation (2 Gy ^137^Cs‐γ‐rays at 1 Gy/min, 24 h post‐injection) daily for 5 days. Silencing of Ape1 in this model translated to a significant increase in overall survival compared to radiation alone.^80^

Docetaxel is another chemotherapy that is commonly used in these nano‐enhancer formulations. Docetaxel causes cells to arrest in the G2/M phase of the cell cycle by acting as an anti‐microtubule agent; cells in this stage are particularly sensitive to radiation.^70^ However, off‐target effects of systematically circulating docetaxel, like those of many chemotherapeutics, are severe in patients and inspire a need for the drug to accumulate specifically at the tumor site.^70^ Researchers encapsulated docetaxel in a PLGA (poly[lactic‐co‐glycolic acid]) NP conjugated with folate to improve tumor targeting for head and neck cancers. This formulation successfully sensitized tumors in mice models more efficiently than free docetaxel and the non‐targeted version of the NPs.^82^ PLGA NPs containing docetaxel have also been used to treat lung and pancreatic cancer in vitro, using d‐alpha‐tocopheryl PEG_1000_ to assist with cellular uptake.^83^ Docetaxel has also been loaded onto titanate nanotubes to treat prostate cancer,^70^ as well as a PCL (polycaprolactone) NP for gastric cancer.^84^ Other chemotherapeutics delivered in nano‐formulations for radiosensitization include paclitaxel,^85^ doxorubicin,^86^ and topotecan.^87^

Another common strategy was to deliver molecules that reduced the effects of hypoxia‐related resistance to radiotherapy. Nitroimidazole is an imaging agent specific to hypoxia; it manages to sensitize tumors to radiation by generating ROS.^88^ Researchers have made “smart” nanogels loaded with IAZA (iodoazomycin arabinoside), a nitroimidazole derivative, and functionalized with galactose. These nanogels were able to sensitize hepatocellular carcinoma (HCC) cells under hypoxic conditions in vitro.^88^ Zong et al. used lipid NPs with encapsulated metronidazoles, a nitroimidazole derivative, and temozolomide, a pro‐drug that releases a DNA alkylating agent, to treat glioblastoma. It successfully increased survival in mouse models when compared to radiotherapy alone.^89^ Another nitroimidazole derivative, liposomal pimonidazole has also been used to radiosensitize melanoma under hypoxic conditions.^90^

Hypoxia‐related resistance to radiotherapy by addressing hypoxia itself, that is, by supplying oxygen to the tumor site. Xu et al. encapsulated perfluorohexane into liposomes because of its high oxygen capacity. This allowed oxygen to be directly administered to the tumor without additional oxygen supply.^91^ They found that the NPs and radiotherapy delayed tumor growth significantly when compared to radiotherapy alone in a mouse model.^91^ A nano‐emulsion of dodecafluoropentane has also been used to increase oxygen in hypoxic tumors in mice, leading to a stronger response to radiation.^92^

### 3.4 Active targeting of nanosized radioenhancers

In an effort to further improve the targeting efficiency, nanosized radioenhancers have been functionalized with moieties able to actively target the tumor, its microenvironment, or the associated vasculature. One common method utilized is by conjugating antibodies to the particle surface. EGFR is a receptor whose over‐expression in cancer cells is linked to cell proliferation, angiogenesis, and tumor metastasis.^93^ One study functionalized gold NPs with anti‐EGFR antibodies, where they were able to sensitize the effects of proton irradiation in cells over‐expressing EGFR but not in cells lacking EGFR.^94^ Another study used anti‐EGFR antibody functionalized to gold NPs to deliver β‐lapachone, an anticancer agent.^95^ These NPs preferentially accumulated in cancer cells according to the amount of EGFR expressed, with higher accumulation occurring in A431s than in A549s, though both had more accumulation in comparison to RKO cells, which lack EGFR. They successfully radio‐sensitized tumors following IV injection in a mouse model with xenografted A549 tumors.^95^ Similarly, both iron‐oxide NPs and silver NPs also have been functionalized to increase sensitivity to radiation for radioresistant glioblastoma and nasopharyngeal carcinoma cells, respectively.^96^, ^97^

In addition, HER2, overexpressed in some cancers, has also been leveraged to improve tumor targeting/treatment of breast, pancreatic, ovarian, endometrial, gastric, and esophageal cancers.^98^ HER2 targeting strategies include monoclonal antibodies (e.g., Traztuzumab, Pertuzumab, etc.), tyrosine kinase inhibitors (e.g., Lapatinib, Neratinib, etc.), Hsp90 inhibitors (e.g., Tanespimycin, Retaspimycin, etc.) or inhibitors of downstream signaling such as mTOR and PI3K pathways (e.g., Everolimus, PI‐103, etc.).^98^ Silica NPs functionalized with hyperbranched polyamidoamine as well as an anti‐HER2 antibody successfully targeted human SK‐BR‐3 breast cancer cells overexpressing HER2.^99^ HER2 is a rational target beyond just breast cancer, as multiple epithelial tumor types correlate HER2 overexpression with poor clinical outcome.^100^ Anti‐HER2 functionalized gold and silver NPs have also been used to radiosensitize breast cancer.^101^, ^102^ Other antibodies used for radioenhancer NPs include Anti‐RhoJ, which is expressed in the vasculature of peri‐ and intratumoral regions, and cmHsp70.1 antibody, which targets a heat shock protein expressed on aggressive glioma cells.^103^, ^104^, ^105^

Another commonly utilized strategy for targeting is to conjugate the particles with folate or folic acid. One study comparing the efficacy of nano‐radiosensitizers decorated with folic acid, glucose, or glutamine found that both glutamine and folic acid significantly increase the efficacy of the radiosensitizers for breast cancer. However, neither showed significant advantage over the other.^106^ Despite this, using folic acid and folate remains a major strategy for tumor targeting nano‐radioenhancers. Combined folate‐ and RBC membrane‐ functionalized bismuth NPs enabled an increased survival in mouse models of breast cancer compared to the nontargeted NPs and radiation alone.^107^ Other studies have used bovine serum albumin NPs with folate to target breast cancer in vitro.^108^ Multiple studies have developed nano‐radioenhancer formulations utilizing folate targeting nasopharyngeal cancer as a model of head and neck cancers. All of these studies demonstrated radio‐sensitizing efficacy using KB cells, which overexpress the folate receptor.^82^, ^109^, ^110^ In vitro studies done by Shakeri‐Zadeh et al. used folate conjugated gold NPs and nanorods to enhance the effects of radiation and photothermal therapy.^109^, ^110^ Werner et al. found that, in vivo, PLGA–lecithin–PEG NPs containing docetaxel with folate were more effective at radio‐sensitizing KB cell tumors than free docetaxel or the nontargeted NPs.^82^ Folate‐conjugated NPs have also been shown to target gliomas for radio‐sensitization, as folate receptors are overexpressed in some brain tumors, as well as on the luminal side of the BBB endothelial cells, which can help bring folate‐conjugated NPs into the brain through the BBB.^111^, ^112^

A variety of other strategies have also been utilized. Conjugating particles with Arg‐Gly‐Asp (RGD) has been used for the radiosensitization of lung, breast, and cervical cancer. RGD peptides recognize a few integrins, including the α_v_β_3_ integrin, which have increased expression on tumor blood vessels and some cancer cells.^113^, ^114^, ^115^, ^116^, ^117^, ^118^ Thio‐glucose is another targeting modality used for a variety of different cancers. Cancer cells exhibit a higher glucose metabolism than normal tissues, resulting in preferential uptake of the thio‐glucose bound NPs than by normal tissue cells.^119^, ^120^, ^121^ In addition, glioma cells, along with the BBB, express low‐density lipoprotein receptor‐related protein‐1 (LRP‐1) which can be targeted with angiopep‐2 conjugated NPs for radio‐enhancement.^89^, ^122^

It is important to note that the majority of cancer nanomedicines that are approved or undergoing clinical trials rely on passive targeting.^123^ In addition, active targeting strategies do not fully address the issue of off target effects^123^ as the targeted receptors are also expressed on normal tissue, though to a lesser extent (e.g., EGFR, HER2, transferrin, folate receptors, etc.).^124^

### 3.5 Radiosensitizer combination for an enhanced effect

Radiosensitizer combinations have also been explored to achieve an enhanced therapeutic effect, including, but not limited to, tandems of: (i) two NP radiosensitizers,^125^ (ii) NP radiosensitizers and a chemotherapeutic drug,^70^ (iii) NP radiosensitizers and tumor oxygenation^126^ or including (iv) dual effect NPs displaying radiosensitizing and glutathione trapping effect.^127^ Indeed, Cheng et al. engineered dumbbell‐like NPs made of gold and titanium dioxide NPs to achieve a synergistic radiosensitization effect in vitro using triple‐negative breast cancer SUM159 cells.^125^ Such technology translated with a significant therapeutic effect both on tumor growth and animal survival in SUM159 tumor‐bearing mice.^125^ Mirjolet et al. have established a synergistic effect of radiosensitizers docetaxel and titanate nanotubes in a murine model of PC‐3 xenografted tumors.^70^ In addition, Song et al. engineered oxygen nanoshuttles made of Bi_2_Se_3_ NPs functionalized with the oxygen carrier perfluorocarbon.^126^ Oxygen is released via the evaporation of perfluorocarbon, triggered by the near‐infrared light activation of the Bi_2_Se_3_ NPs.^126^ Interestingly, Zhang et al. developed “glutathione‐depleting gold nanoclusters” in order to leverage the dual properties of the NPs via both intrinsic radiosensitivity and by sequestering glutathione,^127^ otherwise implicated in ROS “quenching.”^128^

## 4 EARLY CLINICAL TRIALS

There are currently no FDA‐approved nanosized radioenhancers for cancer radiation therapy and a single formulation has received European approval. Clinical trials are evaluating the efficiency and safety of two NP candidates utilizing gadolinium (Gd) chelates into polysiloxane NPs (AGuIX) and hafnium‐based NPs (NBTXR3, also known as PEP503). Polysiloxane Gd‐chelates‐based NPs and hafnium oxide NPs enable both radiosensitization and multimodal imaging of tumors prior to radiation, using MRI and CT, respectively. In trials, AGuIX is administered via intravenous injection (IV), while NBTXR3 can potentially be administered by either intra‐tumoral (IT) or intra‐arterial (IA) routes (NCT01946867 v5 and NCT02721056 v6). The advantage of the IT injection is that it bypasses the challenges associated with inefficient biodistribution to tumors following vascular administration (compared to the initial injected dose).

Clinicaltrials.gov was used to perform the search using the following terms: radiation, cancer, NPs (Table 2). Trials involving NPs used as a mean of drug delivery only were excluded.

### 4.1 Trials involving AGuIX

The first phase I trial (NANORAD, NCT02820454) was dedicated to patients with multiple brain metastasis from non‐small cell lung cancer (NSCLC), breast cancer, colon cancer, or melanoma and therapy consisted of whole brain radiotherapy (WBRT) (10 × 3Gy/fraction over 3 weeks) combined with IV AGuIX nano‐radioenhancers.^129^ The dose escalation was designed with 15, 30, 50, 75, and 100 mg/kg doses.^129^ Fifteen patients were enrolled and no dose‐limiting toxicity (DLT) was observed across the dose escalation cohorts. AGuIX mean plasma half‐life was 1.3 h. Thirteen out of 14 observable patients had stabilization or reduction in tumor burden.^130^ The diagnostic potential of AGuIX was also assessed and compared to Dotarem®, a gadolinium‐based contrast agent.^131^ Results indicate a linear correlation between MRI SE (spin echo) values and increasing AGuIX injected doses. Finally, AGuIX MRI SE was still detected a week post‐injection, which denotes a key improvement in local retention, in comparison to existing Gd‐based contrast agents.^131^

This initial success led to the NANORAD2 phase II trial (NCT03818386) that is currently recruiting patients and consists of 3 × 100 mg/kg AGuIX IV injections (7 days prior to WBRT, before the 1st fraction and before the 6th fraction; 30 Gy and 3 Gy/fraction over 2–3 weeks). The primary endpoint compared to WBRT alone in this randomized trial is assessment of brain disease response at 3 and 6 months using RECIST (Response Evaluation Criteria in Solid Tumors).

A second single arm phase II trial (NANOSTEREO, NCT04094077) aims to assess the efficacy of AGuIX activated by fractionated stereotactic radiotherapy (SRT) in treatment of brain metastasis. Specifically, 100 mg/kg AGuIX is injected intravenously at days 4 and 8 followed by SRT on days 8 to 15 according to standard regimen. Similarly, the primary endpoint is brain metastases' response using RECIST. This trial has been terminated and a new trial design, including a second arm (placebo control group), is currently recruiting (NCT04899908).

Next, the NANOCOL phase I trial (NCT03308604) focused on treating cervical cancer with combined radiotherapy (RT) (45 Gy, 25 fractions over 5 weeks) assisted by AGuIX NPs and cisplatin (40 mg m^−2^ weekly injections during RT), and followed by uterovaginal brachytherapy (15 Gy over 2 weeks).^66^ The study focuses on safety and dose escalation with 20 mg/kg (level −1), 30 mg/kg (level 1), and 50 mg/kg (level 2) doses and follows a modified toxicity probability interval (mTPI) design. Such a clinical trial design has been reported to be safer compared to a standard 3 + 3 phase I design.^132^

AGuIX activated by stereotactic body radiation therapy (SBRT) is also evaluated in comparison with stereotactic magnetic resonance (MR)‐guided adaptive radiation therapy (SMART) alone for the treatment of non‐small cell lung cancer and advanced pancreatic adenocarcinoma (NCT04789486, phase I–II). Phase I will established the recommended phase II dose, while phase II will evaluate efficacy. Primary endpoint is maximum tolerated dose and secondary endpoints include overall response rate, progression‐free survival, overall survival, quality of life data, as well as serious AEs.

Lastly, a phase II trial is currently ongoing with AGuIX activated via hypofractionated proton therapy (NCT04784221). The treatment consists of IV injections of AGuIX on days 1, 8, and 15 and proton therapy (20 fractions, day 1 to 26). The trial is a single arm study that evaluates local efficacy of tumor regression and local progression‐free survival rate.

A new generation of these particles (Bi@AGuIX) is under preclinical evaluation. The formulation includes bismuth (Z = 82) in addition of gadolinium (Z = 64), with the rationale that a higher Z would lead to a greater radiosensitization effect.^133^ This formulation showed superior efficacy in tumor burden in vivo as compared to the radiotherapy control (no particles).^133^ In comparison to the first generation AGuIX, Bi@AGuIX led to a decrease in in vitro cell survival following irradiation.^133^

### 4.2 Trials involving NBTXR3 (also known as Hensify® and PEP503)

The initial phase I study (NCT01433068) focused on locally advanced soft‐tissue sarcoma and involved dose escalation with IT injection of 2.5%, 5%, 10%, and 20% of tumor volume at 53.3 g/L.^134^ This was followed by radiation therapy (5 weeks, 50 Gy, 2 Gy/fraction) starting 24 h following injection, and tumor resection at 6–8 weeks post‐radiation. Dose‐limiting toxicities (DLT) were observed in the 20% dose group and thus the recommended dose was defined at 10% initial tumor volume. This was followed by a randomized, multicentre, international phase II–III trial (NCT02379845) in patients with soft tissue sarcomas. The trial consisted of a single IT injection of NBTXR3 NPs (10% of tumor volume at 53.3 g/L), followed by radiation therapy (5 weeks, 50 Gy, 2 Gy/fraction) starting 24 h following injection, and tumor resection at 5 weeks post‐radiation.^135^ The goal of the study was to determine the DLT and safety profile of NBTXR3. A total of 176 eligible patients were analyzed out of 180 enrolled; 9% of patients developed grade 3–4 AEs; 39% versus 30% of patients developed a serious adverse event (SAE) in the NBTXR3 group versus radiotherapy alone. The primary endpoint was the pathological complete response with a significant difference of 16% versus 8% (*p* = 0.044) in the radiotherapy with NBTXR3 group versus radiotherapy alone. The use of NBTXR3 (Hensify®) in patients with soft tissue sarcomas has been approved in Europe on April 4, 2019.

The success of the initial trials using NBTXR3 led to further clinical trials such as the phase I/II trial (NCT02721056), a dose‐escalation study for patients with HCC or liver metastasis. The dose‐escalation study was designed as follows: 10%, 15%, 22%, 33%, and 42% of tumor volume at baseline, using a 3 + 3 design.^136^ The radiation dose following particle injection was 45 Gy using three fractions of 15 Gy over 5–7 days or 50 Gy using five fractions of 10 Gy over 5–15 days. Interim results published in November 2020 indicated no DLT and a single patient (out of 22) developed a SAE. Disease assessment with RECIST showed five patients with complete response and three with partial response (HCC group), as well as five patients with partial response and one with stable disease (liver metastasis group).^137^ This trial has been terminated following the determination of the recommended phase II dose, along with a change in standard clinical practice for HCC (NCT02721056 v6).

Further translation has been extended to patients with locally advanced squamous cell carcinoma of the oral cavity or oropharynx (NCT01946867 phase I trial). A similar 3 + 3 design was used for the dose‐escalation study (dose levels: 5%, 10%, 15%, and 22% of baseline tumor volume) and IMRT was used to deliver a total dose of 70 Gy in 35 fractions over 7 weeks. Out of 19 patients, five patients developed low‐grade AEs (one grade 1 and four grade 2 events), no DLT and SAE were observed and nine patients had a complete response (out of 13 patients, as measured at 7 weeks following NBTXR3 IT injection).^138^ It is unclear if patients were accrued to the study arm with NBTXR3 IA administration (NCT01946867 version 5) as this arm of the study has not been reported to date.^138^

Additional trials include (i) in advanced cancers (i.e., squamous cell carcinoma of head and neck, metastatic gastric cancer, metastatic cervical cancer, metastatic squamous cell carcinoma, metastatic melanoma, metastatic lung, and metastatic bladder cancers) in conjunction with anti‐PD1 immunotherapy (phase I, NCT03589339) and (ii) in the treatment of prostate adenocarcinoma in conjunction with brachytherapy (phase I/II, NCT02805894). The study design for phase I NCT03589339 assesses IT NBTXR3 activated by stereotactic ablative radiotherapy (SABR) in combination with an anti‐PD1 immunotherapy agent. The study design for phase I/II NCT02805894 included IT NBTXR3 activated by IMRT (phase I) and IT NBTXR3 activated by brachytherapy and IMRT (phase II). This trial has been terminated due to a change in standard clinical practice in the treatment of prostate cancer (NCT02805894 v14).

NBTXR3 is currently being evaluated with additional phase I/II trials. First, a single arm trial is intended for patients with head and neck squamous carcinoma (NCT02901483). The intervention consists of IT PEP503, in combination with weekly administration of cisplatin (dose varies according to PEP503 dose level) and radiation therapy (70–72 Gy, 2–2.12 Gy/fraction over 7 ~ 8 weeks). The primary endpoints of this trial are the determinations of DLT, SAE and the rate of local disease control at 1 year. Further, PEP503 is also being evaluated as radiosensitizer in combination with chemotherapy for patients undergoing neoadjuvant therapy for rectal cancer (NCT02465593). The dose‐escalation study follows a 3 + 3 design and the escalation doses are 5%, 10%, 15%, and 22% of the tumor volume at baseline. The intervention consists of IT PEP503 (day 1), followed by pre‐operative radiation (tumor and nodes: 200 cGy/fraction, 25 fractions; pelvis: 180 cGy/fraction, 25 fractions, 5 times/week) with concomitant chemotherapy: 5‐fluorouracil (225 mg/m^2^ daily, 5 days/week, 5 weeks during radiotherapy) and capecitabine (825 mg/m^2^ twice a day, 5 days/week, 5 weeks during the radiotherapy). Twenty patients were enrolled and distributed as follows for the dose escalation: 7 (5%), 4 (10%), 3 (15%), and 6 (22% of tumor volume at baseline). Data presented at the 2021 American Society of Clinical Oncology Gastrointestinal Cancers Symposium (ASCO‐GI 2021, Poster #66) showed no observed SAEs, highlighting good tolerability of PEP503, and a single DLT (a urinary tract infection). 90% of patients underwent surgery: pathological complete response was measured in 17.6%, and 50% had tumor regression to grade 0 or 1 (using the American Joint Committee on Cancer [AJCC] tumor regression grade [TRG] system). The phase II study is currently ongoing using a 22% PEP503 dose. Next, NBTXR3 is being assessed in the management of locally advanced‐ or borderline‐resectable pancreatic cancer (NCT04484909, phase I). In this single arm study, patients receive IT radioenhancers (day 1), followed by radiation (15 fractions during days 15–43). The primary endpoint is the determination of DLT, MTD and recommended phase II dose. Secondary outcomes include the determination of progression‐free and overall survival (PSS and OS), as well as whether pancreatic injection of NBTXR3 can be achieved. NBTXR3 is also being evaluated for the treatment of inoperable recurrent non‐small cell lung cancer (NCT04505267, phase I). Primary outcomes are the determination of occurrences of DLT and recommended phase II dose. Secondary outcomes include determination of AE, feasibility of injection in lung, lymph nodes, determination of complete, partial response, or stable disease (objective response rate, ORR) along with local disease control rate, and survival parameters.

Recently, NBTXR3 + IMRT is being assessed in conjunction with chemotherapy (NCT04615013, phase I) in the treatment of esophageal cancer. The regimen consists in IT or IN (intra‐nodally) injection of NBTXR3 on day 1 followed by radiation and concurrent chemotherapy (from day 15, 28 fractions over 6 weeks). Primary outcomes are the determination of occurrences of DLT and recommended phase II dose. Secondary endpoints comprise late onset AE, ORR, major pathological response rate, feasibility of injection in tumor and nodes involved, and survival parameters.

Finally, NBTXR3 is evaluated in combination with immunotherapy (pembrolizumab) in the context of recurrent and non‐resectable head and neck squamous cell carcinoma (NCT04834349, phase II). NBTXR3 is administered IT on day 1, followed by SBRT during days 15–29 (first arm) or 15–50 (second arm). Immunotherapy starts on day 15 with repeated cycles every 3 weeks and up to 2 years for both cohorts. Primary endpoints include ORR 6 months post‐RT, progression‐free survival, and late AEs. Secondary endpoints are ORR and overall response at 5 years, acute AEs and survival parameters.

### 4.3 Trial involving Ferumoxytol (superparamagnetic iron oxide NPs)

In November 2020, a phase I prospective observation study has been launched for the use of Ferumoxytol, that is, iron oxide NPs, to enhance radiotherapy using a MR‐Linac in the treatment of primary or metastatic hepatic cancers and liver cirrhosis (NCT04682847, phase I). The MR‐Linac enables both the visualization of the tumor and NPs via MRI, as well as the delivery of radiation therapy.

## 5 CONCLUSION AND FUTURE CHALLENGES

In this review, we discussed the recent advances involving the use of NPs as radiation therapy enhancers, both in preclinical and clinical studies. Such nanoscale technologies still face some critical biological barriers when injected intravenously such as nonspecific biodistribution, clearance by the reticuloendothelial system, hemorheological considerations, and cell internalization.^139^ The use of active targeting strategies has shown promising success (vs. nontargeted nanoformulations) in the context of radioenhancement and drug delivery. However, the vast majority of the injected dose remains inefficiently delivered and is thus cleared. This led some investigators to use the intra‐tumoral route for tumors of known location and, importantly, that are within reach. However, this translates into a limited number of eligible cancers. Thus, the next generation of nano‐radioenhancers would benefit from improved biodistribution and tumor retention. A greater retention might enable a reduction in frequency of injections before each radiation fraction. These efforts could potentially be achieved by combining strategies such as keeping the macrophages in clearance organs occupied prior to nanomedicine injection (such as demonstrated by Germain et al.^34^), and leveraging the shape of NPs for improved targeting and/or retention (such as the strategies described in references,^25^, ^73^ respectively). In addition, approaches leveraging the intra‐operative delivery of nanoformulations post tumor resection would be of particular interest to eliminate potential residual tumor/tumor margins using NP‐sensitized radiation. Such strategy has recently been developed for glioblastoma patients by Grauer et al.^140^ An iron oxide NP paste was applied to the tumor resection site prior to combined hyperthermia and radiation therapies.^140^ A localized inflammatory response was observed and Grauer et al. hypothesized that it might encourage an antitumor immune response. Significantly, two out of six patients exhibited durable responses (overall survival greater than 23 months).^140^

In addition, it would be beneficial to compare multiple formulations in parallel to gain a greater understanding of all the key parameters involved. Furthermore, systematically disclosing the type and energy level of the applied radiation would facilitate the comparison of formulations across trials and studies. An issue that is seen in many studies in the cancer nanomedicine field is different standards of measuring the efficacy of anti‐cancer treatments. Measurement of the efficacy of therapeutics varies in both in vivo and in vitro studies. These differences could be possibly mitigated by reporting the sensitizer‐enhancement ratio, as some studies do, so that formulations' radiosensitizing effect could be better compared across studies. Other considerations would include fully reporting NP's physico‐chemical attributes, including their specific surface. Higher NP surface area directly provides greater opportunities for surface interactions, which is especially important in the context of ROS production.^141^ In this regard, elucidating the specific surface contribution of nano‐radioenhancement by varying size/shape of NPs of similar composition, could help in designing the next generation of nano‐sensitizers.

Numerous preclinical studies regarding the use of NPs as radioenhancers or sensitizers have been published; however, very few studies translate into clinical trials. This could be explained by the difficulty in manufacturing NPs following Good Manufacturing Practices (GMP), along with extensive toxicity studies which are required under Good Laboratory Practice (GLP) conditions. It is worth noting that various routes toward approval can be explored with varied regulatory requirements.^142^ For instance, NBTXR3 (Nanobiotix, France) and AGuIX (NH TherAguix, France) are currently being evaluated as “*medical device*” and “*drug*,” respectively, according to their clinical trial descriptions provided by clinicaltrials.gov.

A further in depth understanding of all the key parameters and specific mechanisms of action involved in NP radiosensitization effects might help address part of the current gap in the translation of these nanotechnologies to the clinic.

## AUTHOR CONTRIBUTIONS

**Colette Bilynsky:** Writing – original draft (supporting); writing – review and editing (equal). **Nadine Millot:** Conceptualization (equal); writing – original draft (supporting); writing – review and editing (equal). **Anne‐Laure Papa:** Conceptualization (equal); writing – original draft (lead); writing – review and editing (equal).

## CONFLICT OF INTERESTS

Authors declare that they have no conflict of interest.

## Data Availability

Data sharing is not applicable to this article as no datasets were generated or analyzed during the current study.
